# Effect of Alginate Coatings on Hydroxyapatite/β-Tricalcium Phosphate Scaffold Behavior

**DOI:** 10.3390/jfb17090461

**Published:** 2026-09-08

**Authors:** Michela Piccinini, Maria Laura Belladonna, Chiara Suvieri, Sara Meoni, Daniela Lanari, Silvia Caponi, Francesco Bonacci, Maurizio Ricci, Alessandro Di Michele, Valeria Ambrogi

**Affiliations:** 1 Department of Pharmaceutical Science, University of Perugia, 06123 Perugia, Italy; michela.piccinini@dottorandi.unipg.it (M.P.); sara.meoni00@gmail.com (S.M.); daniela.lanari@unipg.it (D.L.); maurizio.ricci@unipg.it (M.R.); 2 Department of Medicine and Surgery, University of Perugia, 06129 Perugia, Italy; chiara.suvieri@unipg.it; 3 Istituto Officina dei Materiali, National Research Council (IOM-CNR), Unit of Perugia, c/o Department of Physics and Geology, University of Perugia, 06123 Perugia, Italy; silvia.caponi@cnr.it; 4 Department of Physics and Geology, University of Perugia, 06123 Perugia, Italy; francesco.bonacci@unipg.it (F.B.); alessandro.dimichele@unipg.it (A.D.M.)

**Keywords:** scaffold, hydroxyapatite, β-tricalcium phosphate, alginate, simvastatin, periodontal disease

## Abstract

Chronic oral and maxillofacial diseases are frequently associated with progressive alveolar bone loss, leading to impaired structural integrity of the jaw and increased risk of implant failure, microbial colonization, and microfracture formation. Ceramic scaffolds such as those made of hydroxyapatite (HA) and β-tricalcium phosphate (β-TCP) have attracted considerable attention because of their controlled resorption properties and the promotion of rapid new vital bone formation. The aim of this research is to enhance the performance of scaffolds composed of HA and β-TCP used for guided bone tissue regeneration in oral surgery. HA/β-TCP scaffolds were loaded with simvastatin (SIMV) and coated with multiple layers of alginate (ALG). The proposed strategy was designed to achieve a local and prolonged drug release while simultaneously improving mechanical properties. The scaffolds were characterized in terms of porosity, water absorption, in vitro degradation, in vitro bioactivity, and SIMV release. In addition, microscale mechanical properties were evaluated using Brillouin microscopy before and after the coating process. Cytocompatibility was further evaluated by using murine macrophages as an in vitro cellular model. The results demonstrated that ALG coatings significantly modulated SIMV release, promoting a delayed drug release. Moreover, ALG deposition improved the micromechanical properties of the scaffolds, conferring a dual structure analogous to those of bone tissue. These findings indicate that ALG-coated SIMV-loaded HA/β-TCP scaffolds represent a promising multifunctional platform for the medical field.

## 1. Introduction

Periodontitis is a largely diffused disease characterized by inflammation induced by presence of bacteria. It affects the tooth-supporting tissues and causes alveolar bone resorption which can eventually lead to tooth loss. In addition to periodontal disease, tooth extraction itself may result in significant alveolar bone defects, often compromising the availability of sufficient bone volume for subsequent implant placement. Thus, the development of multifunctional biomaterials capable of promoting bone regeneration while simultaneously exerting anti-inflammatory and antimicrobial effects represents a major challenge in oral and maxillofacial tissue engineering [1].

Materials to be used as cavity fillers for bone repair include metals, ceramics and polymers. Among them, ceramics show several biomedical applications from dental prosthetics to bone regeneration, and some of them are bioresorbable and bioactive [2]. Among ceramics, hydroxyapatite (HA) has long been considered an ideal biomaterial for bone repair because of its chemical composition and high structural similarity to the mineral phase of native bone [3], despite its poor biodegradability. To overcome this limitation, which can hinder the growth of new bone tissue, HA hybrids with the more biodegradable β-TCP have been proposed [4,5].

Even considering their biological advantages, porous HA/β-TCP scaffolds, similarly to other ceramic biomaterials, are intrinsically brittle and exhibit poor mechanical resistance. Consequently, the incorporation of polymeric phases or the application of polymeric coatings has emerged as an effective strategy to improve scaffold mechanical performance and structural stability [6]. Moreover, polymeric coatings may provide further functional advantages by enabling drug loading and local modified release [7], thereby reducing systemic drug distribution and minimizing adverse effects [8,9,10,11].

In this research, a commercially available HA/β-TCP bone substitute for dental implantology (RIGENERA BTK BCP) was functionalized with simvastatin (SIMV) and coated with alginate (ALG) multilayers to evaluate the effects of ALG coatings. SIMV was selected as a model therapeutic agent due to its documented pleiotropic effects on inflammation modulation, bone metabolism, and antimicrobial activity. Moreover, previous studies have demonstrated the potential of SIMV for local administration in periodontitis, and in alveolar sockets to reduce inflammation, to effect bone metabolism and to exert antimicrobial activity against oral bacteria involved in periodontitis [12,13,14,15,16].

SIMV is a prodrug that, when orally administered, is activated into the hydroxyacid form in the liver, where it is mostly retained, resulting in limited extrahepatic bioavailability. Thus, local administration could be a good approach to achieve effects in other districts such as bone and teeth, considering that SIMV activation has been proven also after local administration and in pH > 6 environments [17,18,19,20].

ALG is a natural anionic polysaccharide, widely used in pharmaceutical and biomedical applications. It is extracted from brown algae, which belong to the family of *Phaeophyceae*, or bacteria, such as *Pseudomonas* or *Azotobacter*, and is a linear copolymer composed of 1,4-bound D-mannuronic acid (M) and 1,4-bound L-guluronic acid (G). ALG forms hydrogels when combined with bivalent cations such as Mg^2+^, Zn^2+^ and Ca^2+^, which react with carboxylate groups of the G blocks, forming a three-dimensional network called the “egg box” model. This characteristic makes ALG particularly attractive for biomedical coating and controlled-release applications.

The effects of alginate-based coatings were evaluated in terms of drug release and microscale mechanical properties, the latter being assessed using Brillouin microscopy, an emerging tool in the field of biomechanics [21,22,23].

## 2. Materials and Methods

### 2.1. Materials

Scaffolds composed by 30% of HA and 70% of β-TCP (RIGENERA BTK BCP, hereafter referred to as RIG) were kindly provided by Biotec S.r.l. (Vicenza, Italy). SIMV was purchased from Acofarma (Madrid, Spain). EtOH and ALG (n. 71238, intrinsic viscosity η = 472.02 mL/g and molecular weight 138,836 g/mol [24]) were purchased from Sigma Aldrich Chemical (Milan, Italy). CaCl_2_, NaCl, KCl, MgCl_2_, and Na_2_SO_4_, tris(hydroxymethyl)aminomethane (TRIS) were purchased from Carlo Erba (Milano, Italia). Deionized water was obtained by a reverse osmosis process with a MilliQ system (Millipore, Rome, Italy). Other reagents and solvents were of reagent grade and were used without further purification.

### 2.2. Preparation of SIMV-Loaded RIG Scaffolds

RIG scaffolds (small parallelepiped blocks weighing ca. 40 mg) were immersed in a solution of 50 mg of SIMV in 1 mL of EtOH at room temperature. Then, vacuum was applied for about 5 min to promote the diffusion of the solution into the scaffold pores. The scaffolds were recovered and dried at 37 °C for 24 h (these scaffolds were named RIG/SIMV).

### 2.3. Coating of RIG/SIMV with ALG

A dispersion of sodium ALG (2.5% *w*/*w*) in deionized water was prepared under stirring at room temperature for 12 h until a homogeneous dispersion was obtained.

The dispersion was added dropwise to the RIG/SIMV scaffolds, and then, vacuum was applied for 5 min according to the impregnation method [25,26]. The scaffolds were recovered and dried at 37 °C for about 2 h. Then, they were immersed in a 1.6% calcium chloride solution for about 30 min. The scaffolds were recovered, washed with deionized water, then dried at 37 °C overnight. These scaffolds were named RIG/SIMV/ALG_1_. Scaffolds covered with 2 and 3 coatings (RIG/SIMV/ALG_2_ and RIG/SIMV/ALG_3_, respectively) were obtained by repeating the previously described steps.

### 2.4. Determination of SIMV Loading in Scaffolds

The scaffold (RIG/SIMV and RIG/SIMV/ALG_1_) was weighted and pulverized in a mortar. The obtained powder was put in ethanol (10 mL) and maintained under stirring at room temperature for 24 h. The obtained suspension was centrifuged at 4000 rpm, 20 °C for 10 min, and the concentration of SIMV in the supernatant was then determined by UV–Vis spectroscopy at λ_max_ 239 nm, after proper dilution when necessary.

### 2.5. Characterization

Macroscopic scaffold appearance before and after treatment with ALG was observed by a Nikon ECLIPSE 80i microscope with an E-Plan 10 × 0.25 ocular (Tokyo, Japan). For each sample, diameters of at least 100 pores, randomly chosen, were measured. The evaluation of the pore diameters was elaborated using the “ImageJ” software 1.53v.

FE-SEM photomicrographs were collected through a LEO 1525 ZEISS instrument (Oberkochen, Germany) using Inlens detector and AsB (Angle selective Backscattered) detector. Elemental composition and chemical mapping were determined using a Bruker Quantax EDX (Milano, Italy).

### 2.6. In Vitro Bioactivity Tests in Simulated Body Fluid

Scaffolds were immersed in 5 mL of simulated body fluid (SBF), prepared according to the corrected Kokubo formulation. The solution contained 8.030 g of sodium chloride, 0.354 g of sodium bicarbonate, 0.224 g of potassium chloride, 0.176 g of dipotassium hydrogen phosphate, 0.440 g of magnesium chloride, 0.280 g of calcium chloride, 0.071 g of sodium sulfate, and 6.118 g of tris(hydroxymethyl)aminomethane dissolved in deionized water (q.b. 1000 mL) [27]. The fluid was changed every 7 days, and the test was performed at 37 °C for 28 days. At the end of the test, the scaffolds were recovered from the solution, washed with deionized water, and dried in air.

### 2.7. Water Uptake (WA) and Weight Loss (WL) Measurements

Samples (RIG, RIG/SIMV/ALG_1_, RIG/SIMV/ALG_2_ and RIG/SIMV/ALG_3_) were weighed (W_0_) and then immersed in a flask containing 50 mL of SBF at 37 °C. To measure water absorption (%WA), samples were removed at predetermined intervals, gently buffered to remove excess liquid, and weighed (Wt) [26,28]. %WA was determined using the following equation:

%*W*A = (*W*_t_ − *W*_0_)/W_0_ × 100

Then, to measure the weight loss, samples were dried at 37 °C overnight, then weighed (W_3_). %WL was determined using the following equation:

%*W*L = [(*W*_0_ − *W*_3_)/W_0_] × 100

These experiments were performed for 28 days in triplicate, and results were reported as the average of three measurements ± S.D.

### 2.8. In Vitro SIMV Release from Scaffolds

The scaffolds were immersed in 50 mL of SBF and kept under constant stirring at 30 rpm at 37 °C. The test was conducted in sink conditions. At predetermined time intervals, 1 mL was retrieved and subsequently replenished in equal SBF volume at the same temperature. The SIMV content was determined by UV–Vis spectroscopy (λ_max_ = 239 nm), after proper dilution when necessary. The experiment was performed in triplicate, and the data were reported as the average of three measurements ± SD.

### 2.9. Preparation of SIMV Hydroxyacid Isoform

Hydroxyacid simvastatin sodium salt (SIMV-sodium salt) was obtained from SIMV according to the following literature [29]. To open the lactone ring, 200 mg of SIMV was solubilized in 5 mL of EtOH, then 7.5 mL of NaOH 0.1 N was added. The mixture was heated for 2 h in a closed vessel at 50 °C, yielding the corresponding SIMV-sodium salt. The salt was isolated by removing the solvent with a rotatory evaporator, and the complete conversion of SIMV to SIMV-sodium salt was evaluated by thin-layer chromatography (TLC), nuclear magnetic resonance (^1^H-NMR and ^13^C-NMR) spectroscopy and Ultra High-Performance Liquid Chromatography-Quadrupole Time of Flight (UHPLC–QTOF). TLC experiments were performed on silica gel 60 F_254_ sheets as the stationary phase and ethyl acetate as the mobile phase. Phosphomolybdic acid was used as a detection reagent.

^1^H-NMR and ^13^C-NMR spectra were recorded on a Bruker DRX Advance 400 MH for SIMV and SIMV-sodium salt using DMSO-d_6_ as solvent. Liquid chromatographic separation and mass spectrometric analysis were performed on a UHPLC–QTOF system consisting of an Agilent 1290 Infinity II combined with the Agilent 6560 mass spectrometer (Agilent Technologies Inc., Santa Clara, CA, USA). The chromatographic separation was performed with a ZORBAX RRHD Eclipse Plus C18 column (50 mm × 2.1 mm, 1.8 µm, 95 Å, Agilent Technologies Inc.). UHPLC eluent A was water (LC-MS grade, LiChrosolv, Supelco (Merck, Milan, Italy) with 0.01% (*v*/*v*) formic acid (LC-MS grade, LiChropur, Supelco) and 0.1 mM ammonium formate (LC-MS grade, LiChropur, Supelco), while eluent B was methanol (LC-MS grade, LiChrosolv, Supelco) with 0.1 mM ammonium formate (LC-MS grade, LiChropur, Supelco). The optimized gradient program was as follows: 0–1 min, 20% (*v*/*v*) B; 1–7 min, 20–97% (*v*/*v*) B; 7–12 min, 97% (*v*/*v*) B; 12–13 min, 97–20% (*v*/*v*) B; 13–15 min, 20% (*v*/*v*) B (column equilibration/conditioning). The column temperature was set at 25 °C, and the flow rate at 0.3 mL min^−1^. The injection volume was 5 µL. For MS detection, the Dual AJS ESI source operated in negative ion mode. The gas temperature was set at 300 °C with a flow of 5 L min^−1^, while the sheath gas temperature was 350 °C with a flow of 11 L min^−1^. The nebulizer pressure was set at 35 psi, and the capillary and fragmentor voltages were 3500 V and 400 V, respectively. The Masshunter Workstation Data Acquisition 10.0 (Agilent Technologies Inc., Santa Clara, CA, USA) program was used for data acquisition, while the Masshunter Qualitative Analysis 10.0 (Agilent Technologies Inc., Santa Clara, CA, USA) software was used for data processing.

### 2.10. Brillouin Micro-Spectroscopy (BRMS)

A detailed description of the custom-made setup used to perform the Brillouin micro-spectroscopy measurements is provided in the references [30,31].

In brief, a 532 nm single-mode solid-state laser light is focused by a microscope objective—Mitutoyo M-Plan Apo 20× (Kawasaki, Japan) with a long working distance of 20 mm and numerical aperture (NA) of 0.42. The achieved lateral optical resolution on the samples was about 2 μm. To avoid any photodamage, a laser intensity of about 15 mW on the sample was employed. The quasi-elastic part of the backscattered radiation was analyzed using a Brillouin interferometer (tandem Fabry–Perot TFP-2 HC by JRS Scientific Instruments, Zwillikon, Switzerland). The TFP-2 HC is characterized by a spectral resolution of approximately 0.1 GHz and provides a very high contrast of >150 dB. The setup allows the characterization of material mechanical properties in a non-contact and non-destructive way, with high resolution in both the spatial and frequency domains [32].

The high turbidity of investigated samples is a cause of multiple scattering (MS), which largely affects the Brillouin spectral shape, as it introduces uncertainty in the exchanged wavevector q and thus a loss of defined propagation directionality. In addition, the finite numerical aperture of the microscope objective causes a spread in the collected scattering directions. To obtain a reliable micromechanical characterization, a recently developed fitting procedure was adopted to disentangle these effects from the expected Brillouin line shape of a homogeneous material at a single q [33], which is described by a damped harmonic oscillator function (DHO):

$$I_{DHO}\left(\omega ,q\right)=\frac{I_{0}}{\pi }\frac{\omega_{B}^{2}\Gamma_{B}}{{\left(\omega^{2}-\omega_{B}^{2}\right)}^{2}+{\left(\omega \Gamma_{B}\right)}^{2}}$$

Here, I_0_ is the intensity of the Brillouin peak, while $\omega_{B}$ and $\Gamma_{B}$ are its frequency position and linewidth, related respectively to the real $M^{\prime }=\rho {\left(\omega_{B}/q\right)}^{2}$ and imaginary $M^{\prime \prime }=\omega_{B}\Gamma_{B}\rho /q^{2}$ part of the longitudinal modulus, ρ being the material density. Considering the q dependences of $\omega_{B}=qv_{L}$ and $\Gamma_{B}=D_{k}q^{2}$, where $v_{L}$ is sound velocity of the acoustic waves and $D_{k}$ the kinematic viscosity of the sample, the q-spread due to the objective lens and the loss of q-definition due to multiple scattering can be corrected by splitting the measured peak intensity into ballistic and diffusive contributions. Then, for each contribution, the theoretical DHO intensity is integrated from the minimum exchanged wavevector $q_{min}$, which is respectively set by the collection geometry (NA) and the diffusive nature of the multiple scattered photons (MS), to the maximum value in backscattering configuration $q_{BS}=4\pi n/\lambda$, with λ the incident laser wavelength and n the refraction index of the material. While the low NA = 0.42 objective used here only induces a moderate spread in the collected wavevectors ($q_{min}\approx 0.975q_{BS}$), the contribution from multiple scattering is more important, as all wavevectors from $q_{min}=0$ to $q_{BS}$ contribute to the measured intensity.

### 2.11. Cell Culture of RAW 264.7

The murine macrophage cell line RAW 264.7 was obtained from the American Type Culture Collection (ATCC, Manassas, VA, USA). Cells were maintained under standard culture conditions in RPMI-1640 medium supplemented with 10% heat-inactivated fetal bovine serum (FBS), 2 mM of L-glutamine and antibiotics (100 U/mL penicillin, 100 μg/mL streptomycin). Cell cultures were incubated at 37 °C in a humidified atmosphere containing 5% CO_2_. RIG, RIG/SIMV, and RIG/SIMV/ALG_1_ were selected for preliminary cytocompatibility screening to enable a stepwise comparison of the effects of SIMV loading and ALG coating. RIG/SIMV/ALG_1_ was chosen as the coated formulation because the first ALG layer largely preserved the scaffold porous architecture, whereas additional coating steps resulted in extensive pore occlusion. Prior to biological evaluation, RIG, RIG/SIMV, and RIG/SIMV/ALG_1_ scaffolds were sterilized by UV irradiation for 15 min on each side under aseptic conditions. Subsequently, each scaffold was immersed in 5 mL of complete culture medium and incubated at 37 °C for 24 h. After incubation, the eluate suspensions were centrifuged at 860× *g* for 2 min to remove residual particulate material, and the resulting supernatants, referred to as scaffold eluates (SEs), were collected and used for subsequent in vitro analyses.

### 2.12. Cell Viability Assay

For cell viability experiments, RAW 264.7 macrophages were seeded into 96-well plates at a density of 7.5 × 10^4^ cells/well in 200 μL of complete growth medium and allowed to adhere overnight under standard culture conditions. The following day, the culture medium was removed, and cells were exposed to scalar dilutions of the SEs derived from RIG, RIG/SIMV, and RIG/SIMV/ALG_1_ scaffolds. SE samples were tested either undiluted (1:1) or diluted in culture medium at ratios of 1:2, 1:4, and 1:8. Cells were then incubated for 24 h at 37 °C in a humidified 5% CO_2_ atmosphere [26]. Cell viability was evaluated using the MTT assay (Sigma-Aldrich, St. Louis, MO, USA). Briefly, after treatment, culture supernatants were discarded and replaced with 110 μL of fresh medium containing 50 μg of MTT reagent. Following 4 h incubation at 37 °C, absorbance was measured at 570 nm using a Spark^®^ multimode microplate reader (TECAN, Thermo Fisher Scientific, Waltham, MA, USA). Background controls consisting of MTT-containing medium without cells were included in all experiments. Each experimental condition was analyzed in triplicate.

### 2.13. Statistical Analysis

Cell viability data were analyzed by two-way ANOVA, with scaffold formulation and SE dilution as experimental factors. Dunnett’s multiple-comparisons test was subsequently used to compare RIG/SIMV and RIG/SIMV/ALG_1_ with the corresponding RIG reference group at each SE dilution. Untreated cells were included as a descriptive reference but were not incorporated into the two-way ANOVA because they were not subjected to the SE-dilution factor. Differences were considered statistically significant at *p* < 0.05.

## 3. Results and Discussion

### 3.1. Characterization of the Porous Structure by Optical Microscopy

**Figure 1:**
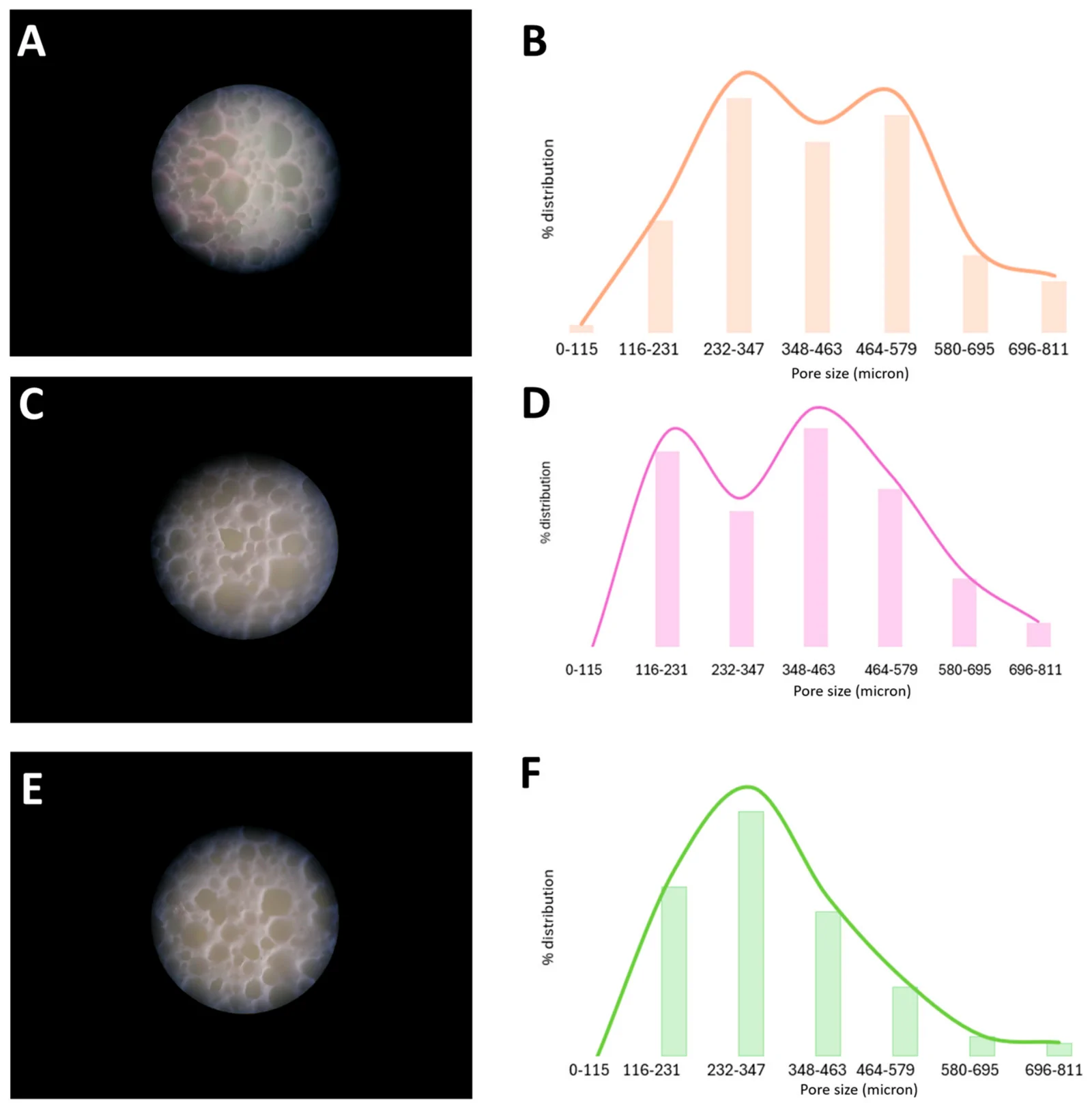
Optical microscopy images (10 × 0.25 resolution) of RIG (**A**), RIG/SIMV (**C**) and RIG/SIMV/ALG_1_ (**E**) and the corresponding pore size distribution (µm) (**B**,**D**,**F**).

The scaffolds RIG, RIG/SIMV and coated scaffolds were analyzed by optical microscopy to evaluate the pore size distribution (Figure 1). In fact, it is reported that the presence of pores and their interconnection are important properties for cell infiltration and vascularization, providing a surface for cell adhesion, migration, proliferation and differentiation [25,26].

In Table 1, the main statistical parameters of the scaffold pore size distribution are reported. Figure 1 highlights how drug loading and subsequent ALG coatings affect the scaffold porosity. Both RIG and RIG/SIMV exhibit a broad pore size distribution with a bimodal behavior (Figure 1B,D). In particular, the presence of SIMV, although it does not alter the bimodal behavior, induces a shift of pore size distribution towards lower values, as a consequence of the drug deposition on the scaffold surface. This is confirmed also by the average diameter of RIG/SIMV, which is equal to 379.6 μm in comparison to the RIG average diameter of 421.0 μm. In RIG/SIMV/ALG_1_, the pores appear mostly rounded, with unimodal and narrower size distributions and a slight decrease in average pore size (Table 1). This result suggests that the first step of treatment with ALG yields only a partial coating of the scaffold, maintaining its porosity (Figure 1E). On the contrary, after the second step, all pores were completely occluded, proving the coating formation (Figure S1). A sufficient pore size for bone regeneration has been reported to be in the range of 200 to 500 μm, which is also ideal for vascularization [25]; thus, the pore occlusion observed for scaffolds with two and three coatings could be a negative aspect, which can be overcome by the progressive degradation of the alginate, successively described (Section 2.3).

The deposition of ALG on the scaffold surface was confirmed by the weight increase of samples, which was 1.93% ± 0.32, 3.67% ± 0.47 and 6.93% ± 0.66 for RIG/SIMV/ALG_1,_ RIG/SIMV/ALG_2_ and RIG/SIMV/ALG_3_, respectively, compared to RIG/SIMV.

SIMV content in 100 mg loaded scaffold was 3.38 mg (drug loading 3.38% ± 0.14), and after the first step, SIMV loading in the RIG/SIMV/ALG_1_ scaffold was 2.25% ± 0.27.

### 3.2. In Vitro Bioactivity Properties of RIG, RIG/SIMV and Coated Samples

**Figure 2:**
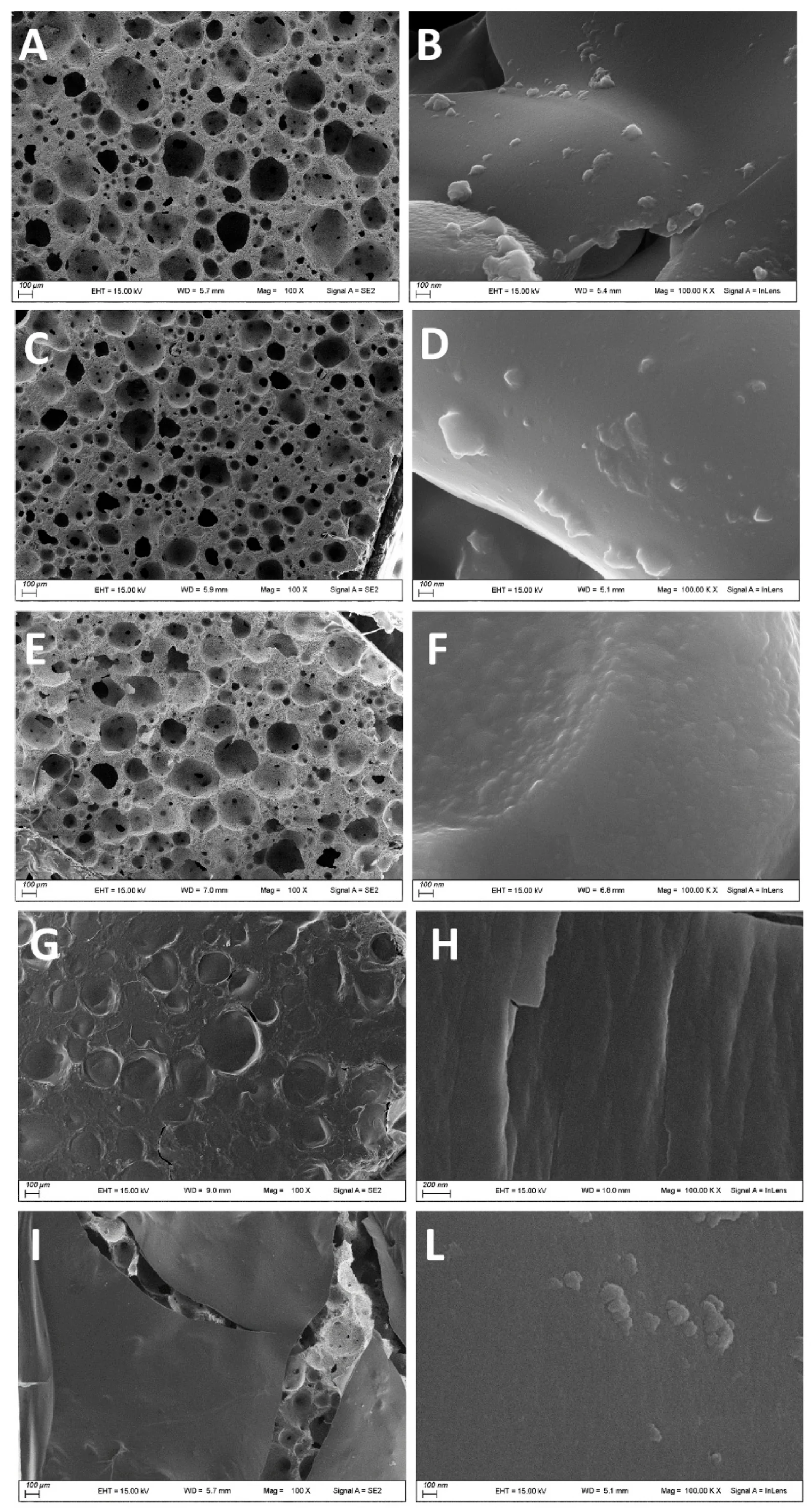
FE-SEM images of RIG ((**A**) at 100 Kx resolution and (**B**) at 100,000 Kx resolution), RIG/SIMV ((**C**) at 100 Kx resolution and (**D**) at 100,000 Kx resolution), RIG/SIMV/ALG_1_ ((**E**) at 100 Kx resolution and (**F**) at 100,000 Kx resolution), RIG/SIMV/ALG_2_ ((**G**) at 100 Kx resolution and (**H**) at 100,000 Kx resolution) and RIG/SIMV/ALG_3_ ((**I**) at 100 Kx resolution and (**L**) at 100,000 Kx resolution) before pre-treatment in SBF.

The in vitro bioactivity was investigated for RIG, RIG/SIMV and coated RIG/SIMV scaffolds to evaluate the role of the scaffold compositions in the formation of new hydroxyapatite when immersed in corrected Kokubo SBF [27]. The samples, after immersion for 28 days, were characterized by FE-SEM. Figure 2 shows SEM images of scaffolds before treatment. Figure 2A–F show macro- and micro-porosity of RIG, RIG/SIMV and RIG/SIMV/ALG_1_ scaffolds. In RIG/SIMV/ALG_2_, the presence of the ALG coating occluding the pore surface can also be observed (Figure 2G,H), and in RIG/SIMV/ALG_3_, the ALG coating appears thicker and fractured (Figure 2I,L).

**Figure 3:**
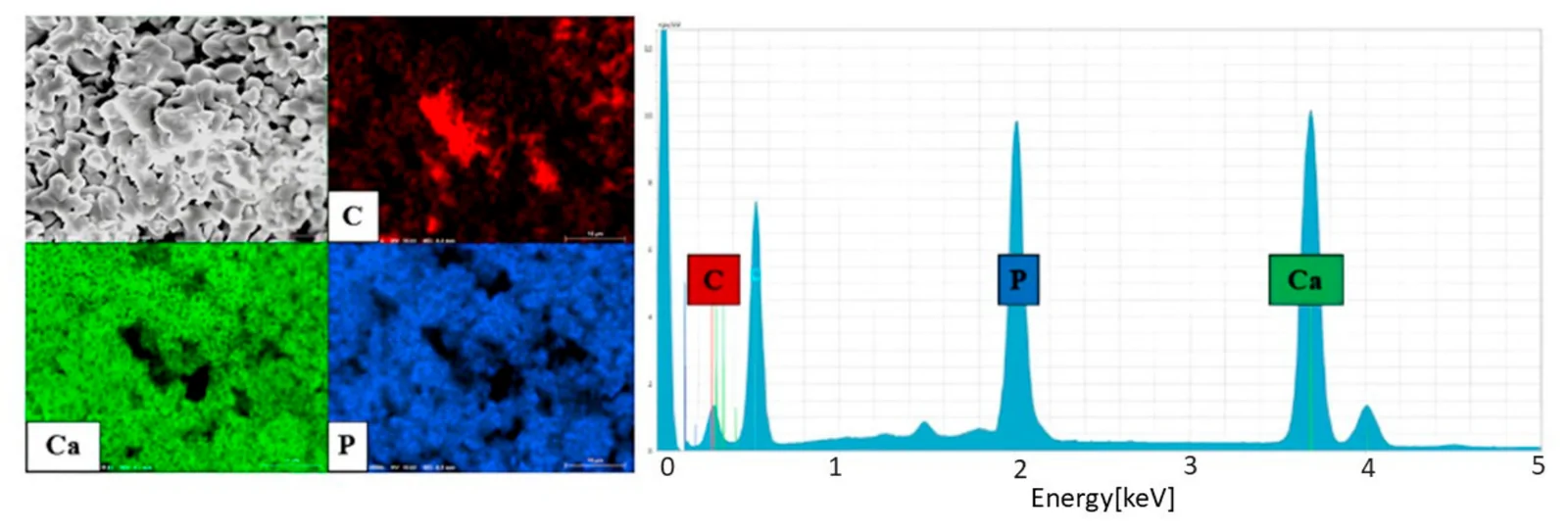
EDX images of RIG/SIMV.

**Figure 4:**
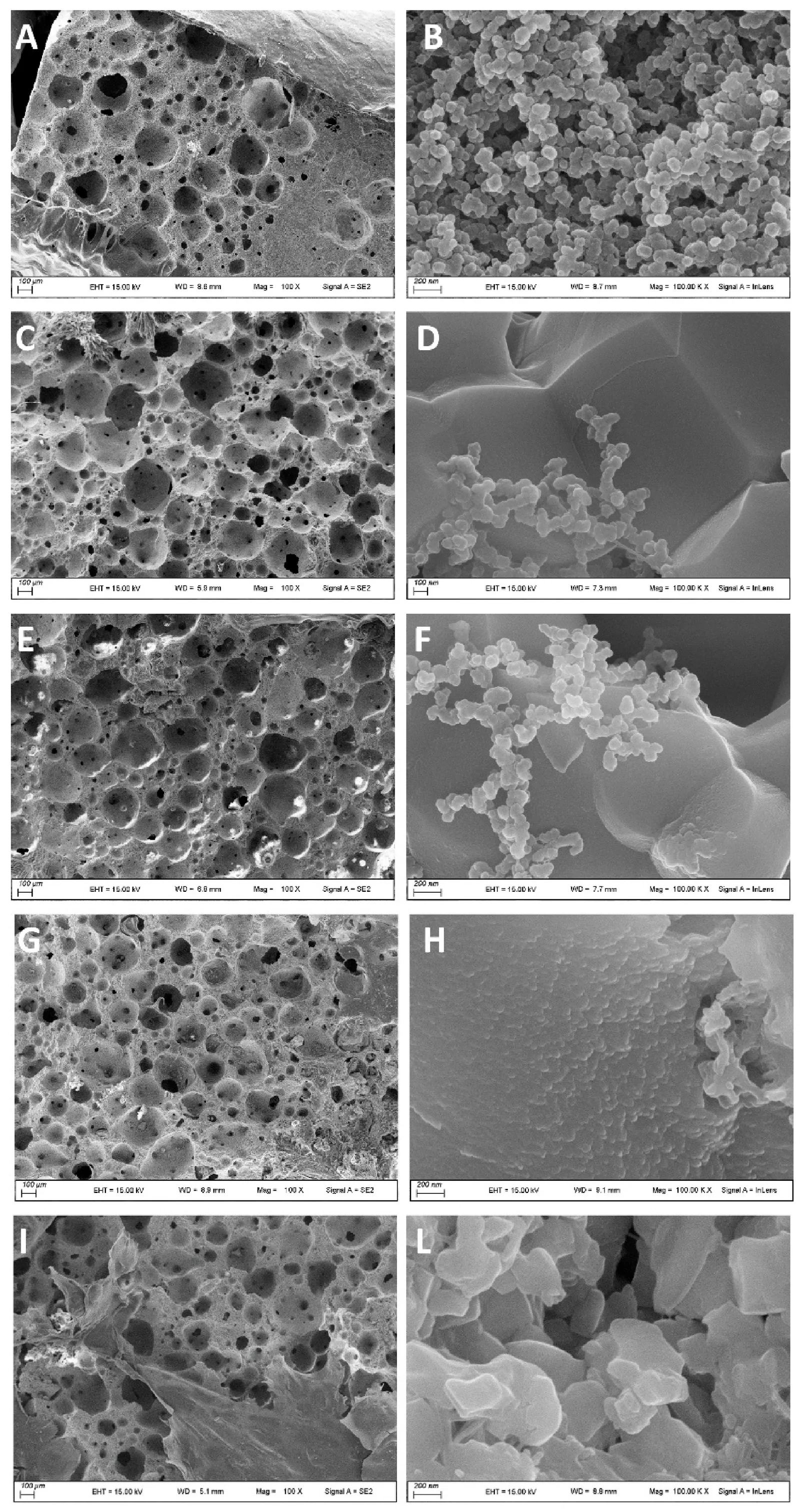
FE-SEM images of RIG ((**A**) at 100 Kx resolution and (**B**) at 100,000 Kx resolution), RIG/SIMV ((**C**) at 100 Kx resolution and (**D**) at 100,000 Kx resolution), RIG/SIMV/ALG_1_ ((**E**) at 100 Kx resolution and (**F**) at 100,000 Kx resolution), RIG/SIMV/ALG_2_ ((**G**) at 100 Kx resolution and (**H**) at 100,000 Kx resolution) and RIG/SIMV/ALG_3_ ((**I**) at 100 Kx resolution and (**L**) at 100,000 Kx resolution) after 28 days in SBF.

The presence of SIMV in scaffold RIG/SIMV was verified by means of elemental carbon mapping performed by EDX. Figure 3 shows the presence of SIMV throughout the scaffold, with higher concentrations in some zones, indicating the presence of aggregates. FE-SEM images of scaffolds recovered after 28 days immersion are reported in Figure 4, where the presence of newly formed spherical crystals on the scaffold surfaces can be observed (panels B, D, F), attributable to HA [26]. This is confirmed by the resulting Ca/P ratio, which was 1.68, typical of HA. In scaffolds RIG/SIMV/ALG_2_ and RIG/SIMV/ALG_3_ also, the FE-SEM images show the presence of newly formed crystals, maybe attributable to HA (Ca/P ratio measured by EDX/microanalysis: 1.68). Figure 4G,I show that the ALG coating was mostly degraded, thus exposing the underlying porous surface. These results can be explained by considering the nature of alginate. Na^+^ ions present in the SBF solution can undergo slow ion exchange with Ca^2+^ ions, inducing a cross-linking decrease and consequently ALG degradation with consequent opening of the pores.

**Figure 5:**
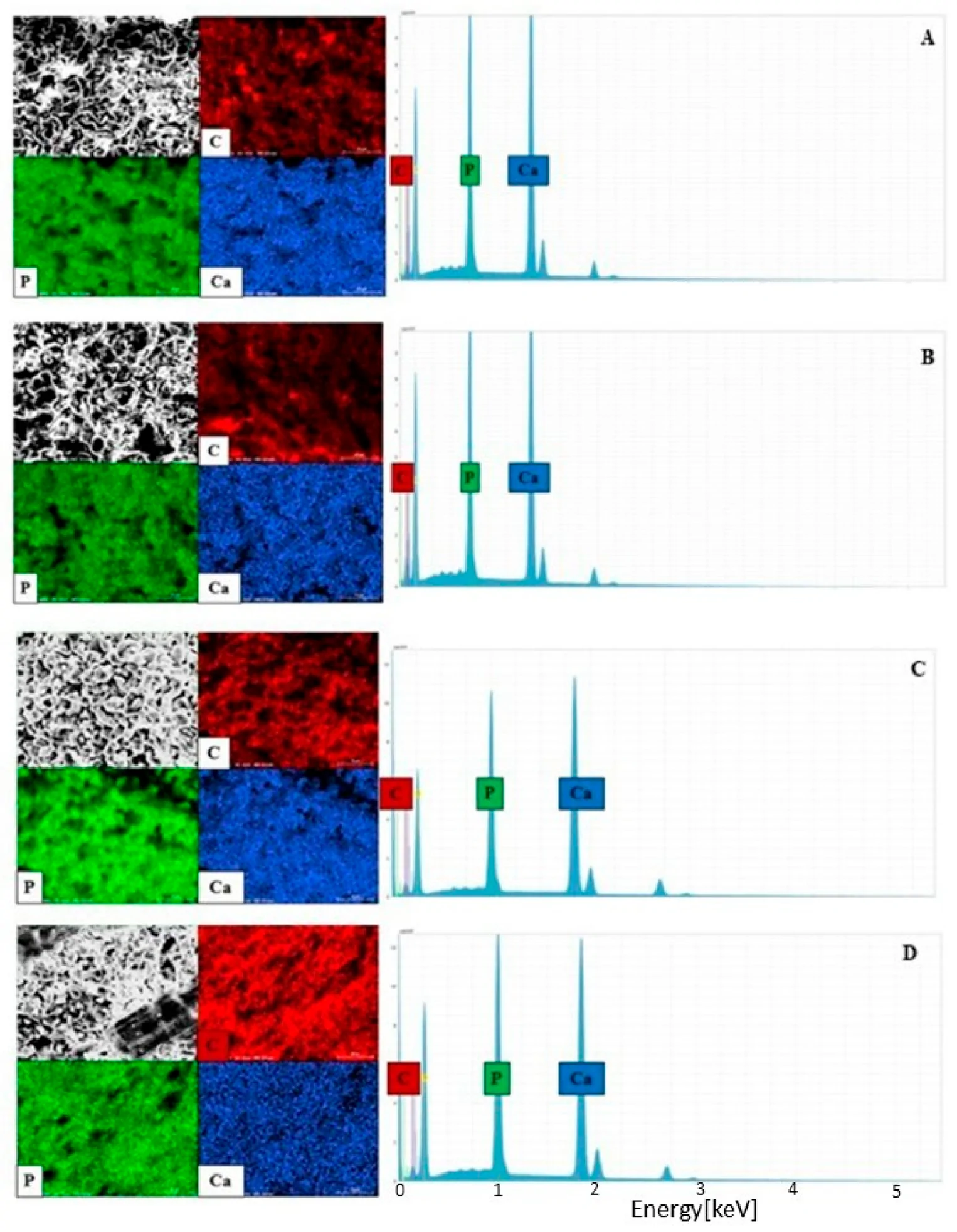
EDX images of RIG/SIMV (**A**), RIG/SIMV/ALG_1_ (**B**), RIG/SIMV/ALG_2_ (**C**) and RIG/SIMV/ALG_3_ (**D**) after 28 days in SBF.

EDX chemical mapping of C, P and Ca of RIG/SIMV, RIG/SIMV/ALG_1_, RIG/SIMV/ALG_2_ and RIG/SIMV/ALG_3_ after 28 days in SBF was performed, and results are reported in Figure 5. The local relative concentration of each element present in the scaffold is indicated by the relative color brightness and intensity.

The presence of carbon, observed in RIG/SIMV also after 28 days (Figure 5A), proves that not all SIMV had been released (see paragraph 3.4). Carbon was present also in the other ALG-covered scaffolds, RIG/SIMV/ALG_1_, RIG/SIMV/ALG_2_ and RIG/SIMV/ALG_3_ (Figure 5B and Figure 5D, respectively), and in this case, it can be attributed to both SIMV and ALG residue.

### 3.3. Water Absorption and Degradation Test

**Figure 6:**
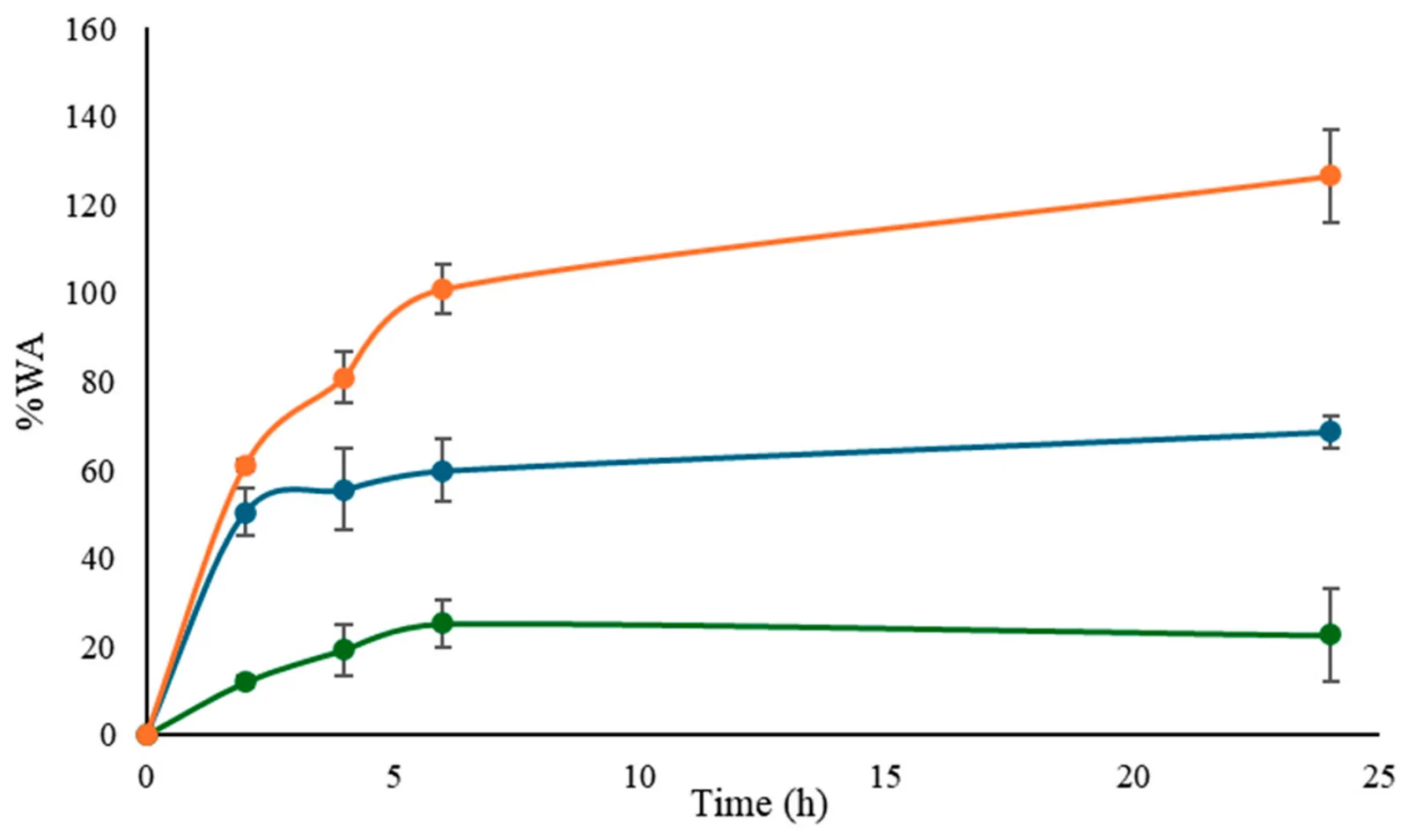
%WA for RIG, RIG/SIMV/ALG_2_ and RIG/SIMV/ALG_3_ after 24 h in SBF.

Scaffolds were monitored for their water absorption, which is one of the properties of polymers used for tissue engineering applications. It reflects the ability of a material to promote cell adhesion, proliferation, and differentiation [26]. In Figure 6, the water absorption capacity of RIG, RIG/SIMV/ALG_2_ and RIG/SIMV/ALG_3_ at predetermined times of immersion in SBF is shown. It has to be underlined that no differences could be observed between RIG and RIG/SIMV and RIG/SIMV/ALG_1_ scaffolds in water absorption. This could be expected, as for RIG/SIMV/ALG_1_, the amount of the ALG coating was so low that it did not affect scaffold hydration.

The results show that RIG/SIMV/ALG_2_ and RIG/SIMV/ALG_3_ experienced greater water uptake than RIG due to the hydrophilic nature of the ALG coating and to its swelling ability. Moreover, the figure indicates that the majority of water uptake occurred within the first 6 h of immersion, reaching approximately 20%, 50% and 140% WA for RIG, RIG/SIMV/ALG_2_ and RIG/SIMV/ALG_3_, respectively. These results can be explained by considering the material composition, hydrophilicity and porosity of the samples.

Scaffold degradation plays a key role in the overall regeneration process, as it influences the balance between material resorption and new bone deposition. Under simulated physiological conditions, β-TCP degrades more rapidly than HA. This results in the supersaturation of calcium and phosphate ions in the surrounding microenvironment, a condition that promotes the precipitation of new apatite crystals [34]. Thus, the weight variation (%) for the scaffold immersed in SBF was evaluated, and results are shown in Table 2.

As reported in Table 2, all scaffolds showed moderate weight loss after 28 days, as a result of two opposite phenomena: β-TCP and ALG degradation and growth of new hydroxyapatite [23,26,33].

In particular, the presence of alginate determines, as expected, lower in vitro bioactivity in comparison to uncoated scaffolds, a parameter which has to be carefully evaluated for their possible use in bone regeneration.

### 3.4. In Vitro Release of SIMV from Scaffolds

**Figure 7:**
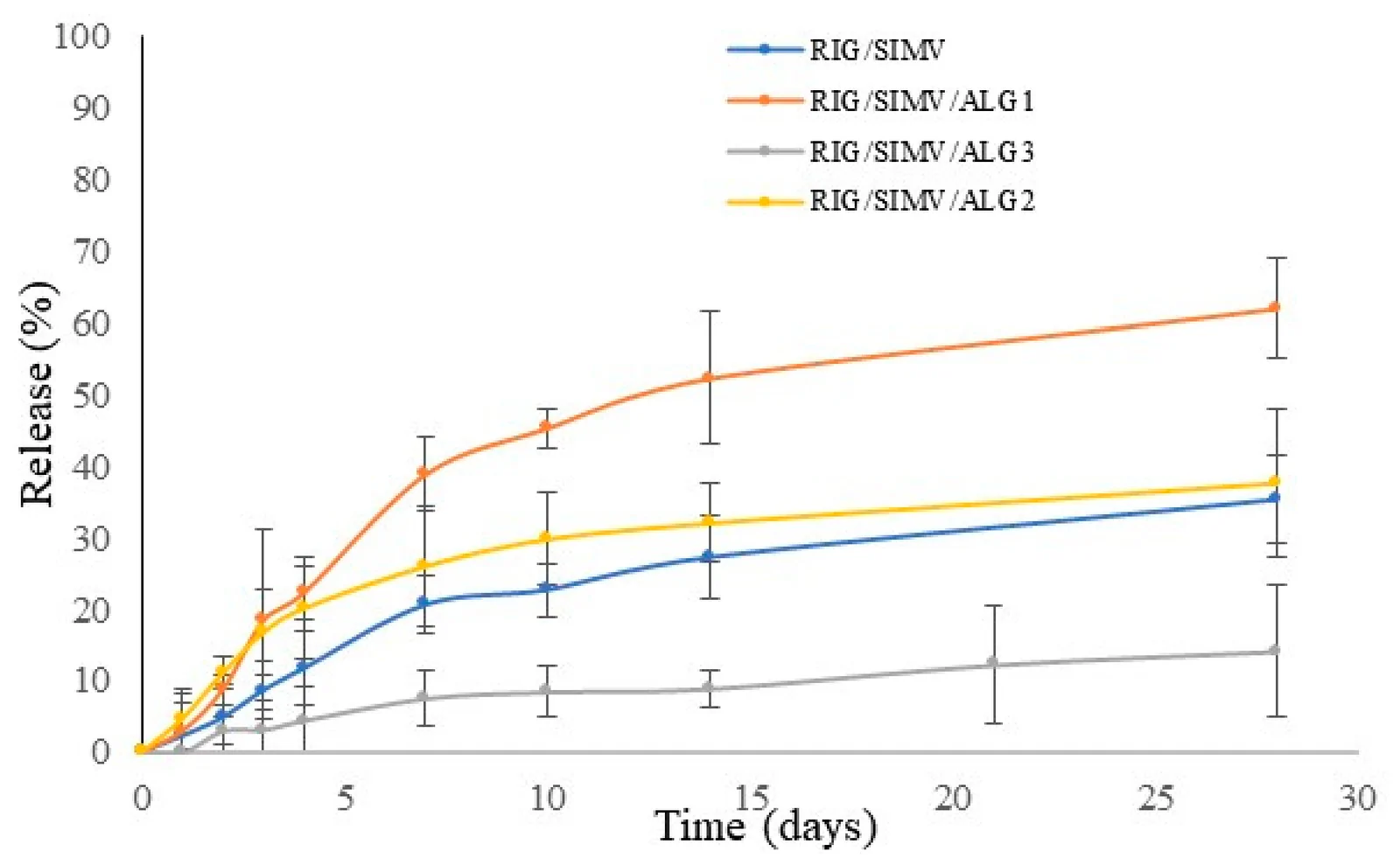
In vitro SIMV release (%) from scaffolds of RIG/SIMV, RIG/SIMV/ALG_1_, RIG/SIMV/ALG_2_ and RIG/SIMV/ALG_3_.

SIMV release from the scaffolds was evaluated in SBF. Results are reported in Figure 7 and expressed as percentage release as a function of time. The SIMV release was very slow from all scaffolds. The cumulative amount of SIMV released after 28 days was approximately 36% for RIG/SIMV, 62% for RIG/SIMV/ALG_1_, 38% for RIG/SIMV/ALG_2_, and 15% for RIG/SIMV/ALG_3_. This incomplete mass balance is consistent with the very slow diffusion of SIMV through the scaffold and ALG coating, and indicates that a fraction of the SIMV remains available for release beyond the 28-day observation window. As reported in Figure 7, after 28 days, no plateau could be reached. Differences could be observed due to the presence of the ALG coatings. Upon immersion in SBF, ALG undergoes hydration within 6 h, forming, around the scaffold, a highly viscous hydrocolloid layer which must be crossed by the drug molecules to be released; therefore, the greater the coating thickness, the longer the diffusion path length [21,34]. However, this behavior was observed only for RIG/SIMV/ALG_3_ by comparing the release patterns from the scaffolds as the number of ALG layers increased. Surprisingly, only the SIMV release from RIG/SIMV/ALG_3_ was slower than that from the uncoated scaffold, while the resulting release from the uncoated scaffold (RIG/SIMV) was lower with respect to both RIG/SIMV/ALG_1_ and RIG/SIMV/ALG_2_. The slow SIMV release can be due to its high lipophilicity and low solubility. The unexpected behaviors of RIG/SIMV/ALG_1_ and RIG/SIMV/ALG_2_ may be due to the presence of the low amount of hydrophilic ALG, which improves drug release getting better the wettability of the poorly water soluble SIMV, leading to a reduction of its agglomeration [34,35]. When the amount of ALG was increased (RIG/SIMV/ALG_3_), SIMV release decreased and a one-day lag time was observed. In fact, in RIG/SIMV/ALG_3_, the ALG coating was thicker (Figure 2I); it absorbed a greater amount of water (Figure 7) and formed a viscous gel around the scaffold, through which SIMV diffused very slowly. The slow SIMV release—with no plateau reached even after 28 days—is beneficial, as SIMV acts at various stages of the bone regeneration process, ranging from the inflammatory phase to vascularization and bone metabolism; furthermore, the slow release prevents high concentrations that could prove harmful and compromise bone repair [13,14,15,35].

Overall, these findings demonstrate that the number of ALG coating layers can modulate the SIMV release profile. The intended function of the ALG coating was to achieve a sustained and tunable release profile by modulating drug–medium interactions and diffusion from the scaffold. However, the optimal release concentration cannot be defined independently of the biological endpoint and experimental model. The present study therefore establishes the ability of the coating to modulate SIMV release, whereas dedicated dose–response studies in osteogenic, immunological, and microbiological models will be required to identify the corresponding biologically effective concentration ranges.

In view of the existing literature, SIMV hydrolysis could be expected under the conditions of our experiments at pH 7.4 and 37 °C [20]. Furthermore, the scaffolds here described are for a local application, and therefore, it can be hypothesized that local esterases are able to hydrolyze the prodrug, once released, promoting its activation [18,19,20]. To evaluate the stability of SIMV in the conditions of biological in vitro tests, it was considered appropriate to assess the nature of the released SIMV (lactone or hydroxy acid form) from the scaffold. To gain insight into this aspect, SIMV sodium salt was synthesized as previously described in paragraph 2.10, and its structure and purity were verified by NMR spectroscopy and UHPLC.

Successively, the scaffold RIG/SIMV and SIMV (powder) were immersed for 1 day in 5 mL of SBF, and then the release medium was analyzed by UHPLC.

Table 3 shows that after 1 day of immersion in the release medium, the presence of SIMV-sodium salt was detected in both samples (RIG/SIMV and SIMV). The peak height (SIMV-sodium salt/SIMV) ratios were 5.68 and 0.513, respectively, for RIG/SIMV and SIMV, meaning that the peak height for SIMV-sodium salt in sample RIG/SIMV was 11 times higher than that obtained from neat SIMV. Thus, the hydrolysis of SIMV was higher when SIMV was adsorbed on the scaffold. This can be explained by the fact that SIMV is a hydrophobic molecule, resulting in poor wettability of the SIMV particles suspended in SBF and thus in reduced hydrolysis. On the contrary, in the scaffold, SIMV is widely distributed on its surface and therefore has a high surface area of contact with the fluids, which consequently allows the possibility of greater molecular hydrolysis. Moreover, it could not be excluded that the presence of a more alkaline microenvironment on the scaffold surface, due to the exchange of ions with the ions of SBF, could favor SIMV hydrolysis.

### 3.5. Mechanical Properties

Bones are heterogeneous tissues made of soft and hard micro-structures that ensure the correct tissue functionality. The presence of a soft part is associated with the organic component of the tissue (collagen fibers, cells, and Haversian and Volkmann’s canals) and guarantees the exchange of nutrients and supports bone regeneration. This soft component is interspersed with the hard structures associated with the mineralized fibers, proving the bone tissue with rigidity and the ability to withstand stress [36].

**Figure 8:**
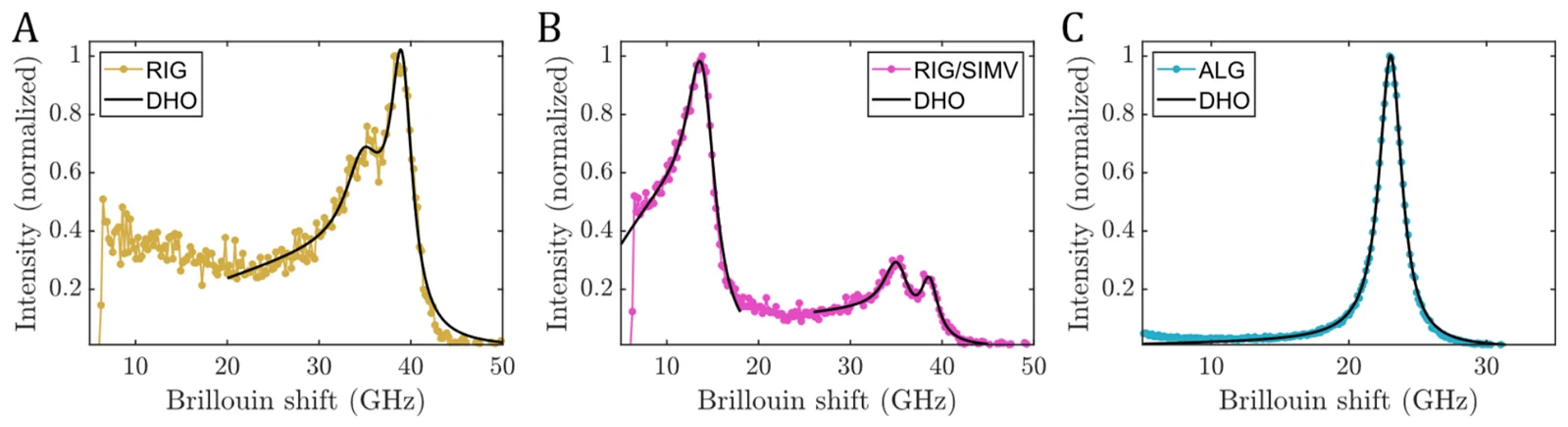
Brillouin spectra in low- and high-frequency regions for RIG (**A**), RIG/SIMV (**B**) and ALG (**C**).

Brillouin microscopy is an all-optical and contactless technique that can provide information on the mechanical properties of materials at the microscale. In previous studies on bone samples, indeed, Brillouin spectroscopy revealed the co-presence of at least two distinct main spectroscopic features, relative to the coexistence of these soft and hard components [37,38]. Figure 8 shows Brillouin spectra in the low- and high-frequency regions for RIG (A), RIG/SIMV (B) and ALG (C).

The peaks at 35. 5 and 39 GHz (Figure 8A) suggest the presence of two distinct components of micrometric size with similar rigidities. Note that a single DHO function, even if multiple scattering is considered explicitly in the fitting procedure (see Materials and Methods), often fails to fit properly the RIG peak. The intensity ratio between the peaks at 35.5 and 39 GHz varies slightly across different sample points (compare, for instance, the high-frequency regions of panels A and B in Figure 8), indicating some compositional variations within the RIG phase. In particular, the high frequency peak at 39 GHz is consistent with the known longitudinal velocity $v_{L}\sim 6.5\;km/s$ of ceramics composed of HA and β-TCP, with HA as a predominant phase [39]. In fact, assuming a refractive index of 1.60 for the scaffold (https://webmineral.com/data/Carbonate-hydroxylapatite.shtml accessed on 31 August 2026), from the measured Brillouin shift, we obtain $v_{L}=6.48\;km/s.$ The other peak at 35.5 GHz could be associated with the presence of calcium oxide impurities, maybe due to the sintering process, which are known to lower the sound velocity to about 6.0 km/s [39]. Since CaO occurs preferentially on the fracture surfaces, this could also explain the variability in the intensity ratio of the RIG peaks.

When the scaffolds are functionalized with SIMV, a second peak appears in the spectrum at around 14.5 GHz, see Figure 8B, indicating a significantly softer response. Brillouin measurements on pure ALG (Figure 8C) exhibit instead a rigidity value intermediate between the SIMV and RIG phases, as evidenced by the peak at approximately 23 GHz. Differently from RIG, for both ALG and SIMV phases, a single DHO function is sufficient to capture the peak shape, indicating that these compounds are effectively homogeneous at the probed length and time scales.

Table 4 summarizes the average peak positions and linewidths of the three components across several points in the scaffolds with and without ALG coating (mean ± standard deviation). The pronounced width of the RIG peak can be associated with the porous microscopic structure of the material, which induces a phonon scattering process and consequent attenuation [40].

The large spectral differences between pure RIG, ALG and SIMV allow us to identify the presence of these three components on the scaffold surface.

**Figure 9:**
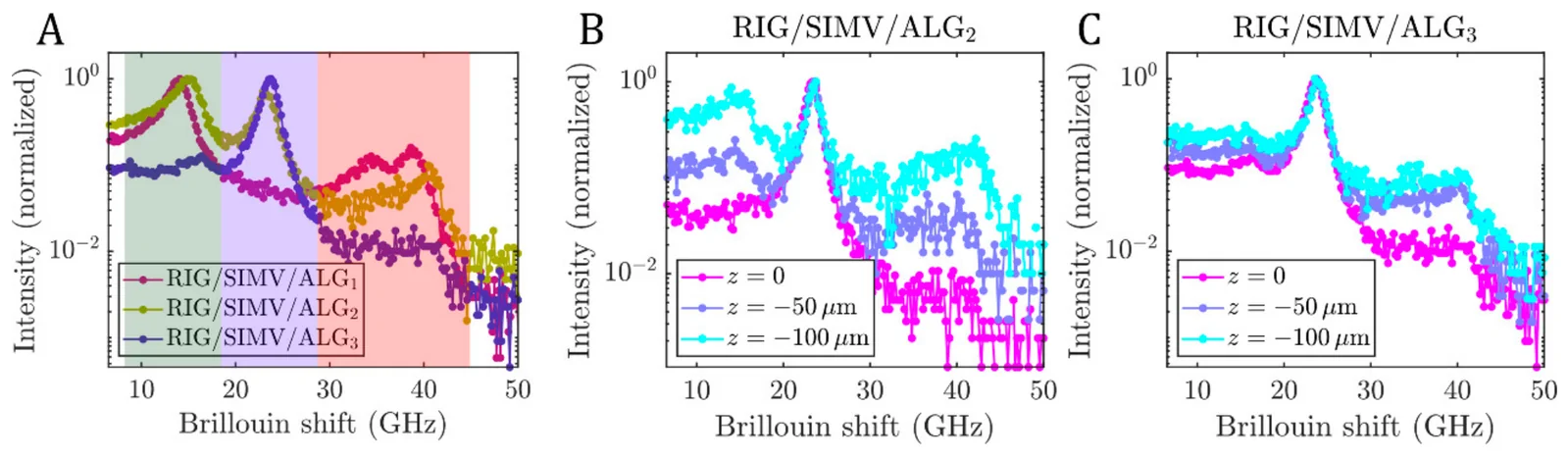
(**A**) Spectra acquired on the RIG/SIMV/ALG_1,_ RIG/SIMV/ALG_2_ and RIG/SIMV/ALG_3_ surfaces and (**B**,**C**) spectra acquired at different depths from the surface of RIG/SIMV/ALG_2_ and RIG/SIMV/ALG_3_, respectively.

In Figure 9, spectra acquired on coated scaffolds are reported. As shown in Figure 9A, in the scaffolds functionalized with SIMV and covered with a single coating of ALG (RIG/SIMV/ALG_1_), the Brillouin measurements display the typical peaks of both SIMV of bare RIG in the low- and high-frequency regions, respectively, but there are no peaks attributable to ALG, maybe due to a low amount of ALG or insufficient ALG coverage.

In the scaffold with SIMV and covered with a double or triple coating of ALG (RIG/SIMV/ALG_2_ and RIG/SIMV/ALG_3_), instead, the peak attributable to ALG at 23 GHz is clearly present and dominates the spectra. The peaks attributable to SIMV (14.5 GHz) and RIG (35.5 and 39 GHz) are also present, although their intensity is reduced compared to bare SIMV/RIG scaffolds and depends on the sampled points. A possible explanation of this effect can be ascribed to a reduced penetration of the excitation laser below the thicker ALG coating. Also, variability in the relative peak intensities across the same sample may be due to heterogeneous ALG coating and/or to the presence of significant surface roughness.

To further assess the ALG thickness, we also performed a z-scan, acquiring BLS spectra at different depths from the sample surface. Figure 9B,C show typical vertical scans respectively obtained in the RIG/SIMV/ALG_2_ and RIG/SIMV/ALG_3_ samples, using a step ∆z=50 μm. Here, the Brillouin intensity is normalized to the ALG peak at 23 GHz. As evident, going deeper into the samples results in a decrease in the peak associated with ALG, while the two RIG peaks (35.5 and 39 GHz) increase. As the peak intensities are primarily related to the amount of each component within the scattering volume, this trend indicates that the ALG contribution decreases with depth, consistently with a layered architecture in which ALG mainly covers the scaffold surface. At the same time, we observe a concomitant degradation of the Brillouin signal, caused by increased multiple scattering in these non-transparent scaffolds. This reduction in SNR, together with surface and coating heterogeneities, hampers a more quantitative characterization of the ALG thickness. In fact, in our scaffolds, the soft (ALG) and hard (RIG) phases form a layered architecture resulting from the successive coating process, with an opaque ALG layer deposited on top of the RIG/SIMV substrate. Consequently, optical access to the underlying RIG phase depends on the opacity and thickness of the ALG coating.

### 3.6. Cytocompatibility Assessment of SIMV-Loaded and ALG-Coated Scaffolds

The cytocompatibility of scaffold-derived eluates was evaluated using RAW 264.7 murine macrophages in order to investigate the potential biological effects associated with the release of SIMV and alginate-derived components from the functionalized HA/β-TCP scaffolds. Since macrophages play a pivotal role in the inflammatory phase of bone healing and in the host response to implanted biomaterials [41], the assessment of macrophage viability represents an important preliminary indicator of scaffold biocompatibility. The concentration of SIMV released into FBS- and antibiotic-free medium after 24 h was quantified by UV–Vis spectrophotometry.

The nominal total SIMV concentrations measured in the undiluted RIG/SIMV and RIG/SIMV/ALG_1_ SEs were 35.2 ± 10.0 and 44.5 ± 2.1 µM, respectively. The SEs were tested either undiluted or at dilution ratios of 1:2, 1:4, and 1:8 to evaluate cell viability across a range of SIMV exposure levels.

**Figure 10:**
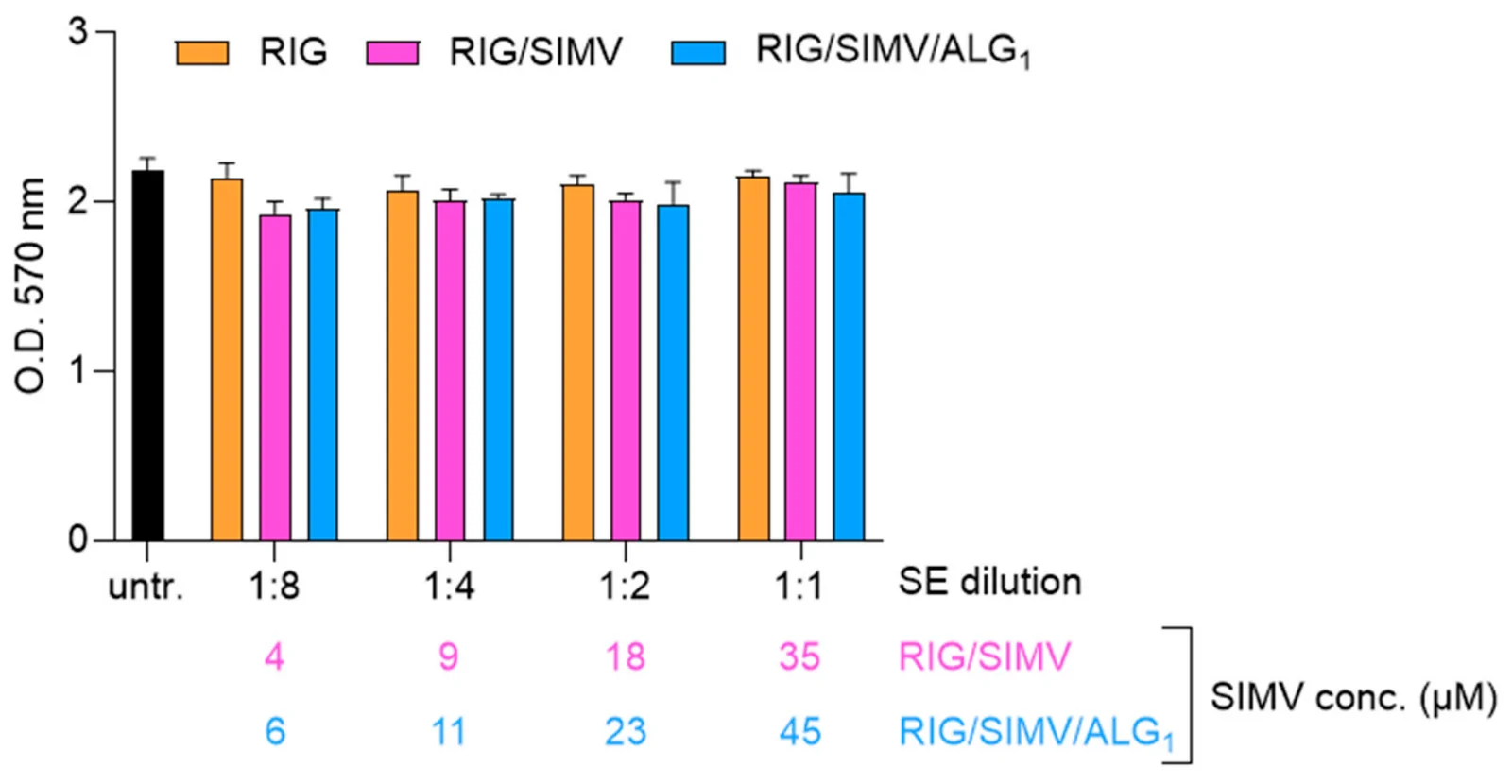
MTT analysis of cell viability in cultures of RAW 264.7 cells treated with SEs. Cell viability following treatment with RIG, RIG/SIMV, or RIG/SIMV/ALG_1_ SE at the indicated dilution ratios was evaluated using the MTT colorimetric assay. The final concentration of SIMV in cultures treated with the different SE dilutions is indicated in magenta and light blue for RIG/SIMV and RIG/SIMV/ALG_1_, respectively. Data are presented as the mean ± SD of three replicates. Differences between RIG/SIMV or RIG/SIMV/ALG_1_ and the corresponding RIG reference group were analyzed by two-way ANOVA followed by Dunnett’s multiple-comparisons test. No statistically significant differences were detected in any of the comparisons (*p* > 0.05).

RAW 264.7 cells were exposed for 24 h to SEs obtained from RIG, RIG/SIMV, and RIG/SIMV/ALG_1_ scaffolds at different dilution ratios (Figure 10). Cell viability remained generally consistent across the tested formulations and SE dilutions, and none of the eluates caused a marked reduction in viability under the experimental conditions employed. Two-way ANOVA followed by Dunnett’s multiple-comparisons test showed no statistically significant differences between RIG/SIMV or RIG/SIMV/ALG_1_ and the corresponding RIG reference group at any of the tested SE dilutions (*p* > 0.05 for all comparisons). Descriptively, the viability values measured after exposure to RIG-derived SEs were close to those observed in untreated cells. Overall, these findings indicate that neither SIMV loading nor the addition of a single ALG coating produced a detectable reduction in RAW 264.7 cell viability relative to the corresponding unmodified RIG scaffold eluates.

Since RIG/SIMV/ALG_2_ and RIG/SIMV/ALG_3_ were not included in the present cytocompatibility assay, no conclusions regarding their biological tolerance can be drawn from these results. Their biological evaluation will therefore be required before selecting the optimal coating configuration.

Although SIMV is known to exert dose-dependent cytotoxic effects at elevated concentrations [42], no significant reduction in RAW 264.7 cell viability was observed at the highest nominal SIMV concentrations tested, corresponding to 35.2 and 44.5 µM in the undiluted RIG/SIMV and RIG/SIMV/ALG_1_ SEs, respectively. This finding is consistent with the study by Nguyen et al., who reported no significant reduction in RAW 264.7 cell viability following 48 h exposure to SIMV at 20 µg/mL, corresponding to approximately 47.8 µM [42]. More generally, the cellular response to SIMV may vary depending on cell type, exposure duration, drug formulation and chemical form, culture-medium composition, and the fraction of drug that is freely available to cells [42,43,44]. Accordingly, the present findings apply specifically to RAW 264.7 macrophages exposed for 24 h to scaffold-derived eluates and should not be interpreted as defining a general non-cytotoxic concentration threshold for SIMV. Notably, eluates derived from RIG/SIMV/ALG_1_ scaffolds exhibited cytocompatibility comparable to that observed for both uncoated RIG/SIMV and pristine RIG scaffolds. These findings indicate that neither SIMV loading nor the presence of a single ALG coating adversely affected cell viability under the experimental conditions adopted. The maintenance of a comparable cellular response among all scaffold formulations demonstrates that the coating process did not compromise the biological performance of the ceramic substrate. Furthermore, ALG is a well-established biocompatible polysaccharide widely employed in tissue engineering and drug delivery applications, and its incorporation is consistent with the preservation of a favorable microenvironment for cell survival [26,45].

Overall, under the extract-based experimental conditions employed, eluates obtained from RIG, RIG/SIMV, and RIG/SIMV/ALG_1_ scaffolds did not cause a marked reduction in RAW 264.7 macrophage viability, providing preliminary evidence that SIMV loading and the first ALG coating do not introduce detectable cytotoxic effects at the tested eluate concentrations. However, this assay does not assess direct cell–material interactions, immunomodulatory responses, or the osteogenic potential of the scaffolds. Further studies using direct cell seeding, osteoblasts or mesenchymal stromal cells, and three-dimensional culture models will therefore be required to evaluate cell adhesion, infiltration, proliferation, osteogenic differentiation, and matrix mineralization. Accordingly, the present findings should be regarded as a preliminary cytocompatibility basis for the subsequent biological evaluation of these scaffolds in bone regeneration models.

## 4. Conclusions

This study provides a physicochemical characterization of SIMV-loaded HA/β-TCP scaffolds coated with increasing numbers of ALG layers. SIMV was successfully incorporated into the ceramic scaffold, while ALG deposition modified its porous architecture, water uptake, degradation behavior, drug-release profile, and micromechanical properties. In vitro immersion in SBF resulted in the formation of newly precipitated hydroxyapatite, although increasing ALG deposition also progressively affected scaffold porosity. The effect of ALG on SIMV release was dependent on coating thickness: the first two coating steps promoted drug release, likely by improving scaffold wettability, whereas the thicker three-layer coating produced a more pronounced diffusion barrier and delayed release. Brillouin microscopy highlighted the micromechanical contributions of the ceramic matrix, SIMV, and ALG and showed that the two- and three-layer coatings generated a soft detectable ALG-associated surface component. These findings demonstrate that the number of ALG layers can modulate several physicochemical properties of SIMV-loaded HA/β-TCP scaffolds, while also highlighting the need to balance coating thickness, pore preservation, and drug-release behavior when selecting the most appropriate formulation. The biological evaluation was limited to a preliminary extract-based cytocompatibility assay using RAW 264.7 macrophages. Under the investigated conditions, eluates from RIG, RIG/SIMV, and RIG/SIMV/ALG_1_ did not cause a marked reduction in cell viability. However, these results cannot be extrapolated to the two- and three-layer coated formulations and do not demonstrate direct cell–material compatibility, immunomodulatory activity, or osteogenic efficacy.

## Figures and Tables

**Table 1:** Main statistical parameters of the scaffold pore size distribution.

Scaffolds	Average Diameter (μm) ± SD	Mode (μm)	Median (μm)
RIG	421.0 ± 160.0	322.0 509.0	409.0
RIG/SIMV	379.6 ± 158.7	127.0 347.0	381.0
RIG/SIMV/ALG_1_	333.3 ± 139.1	289.0	312.0

**Table 2:** %WL for scaffolds after 28 days in SBF.

Sample	WL After 28 Days ± S.D. (%)
RIG	−3.01 ± 0.67
RIG/SIMV	−0.07 ± 1.34
RIG/SIMV/ALG_1_	−2.63 ± 0.83
RIG/SIMV/ALG_2_	−4.22 ± 0.23
RIG/SIMV/ALG_3_	−3.30 ± 1.80

**Table 3:** Peak height of SIMV-sodium salt and SIMV and their ratio after 1 day release.

Samples	Time (Days)	Peak Height SIMV-Sodium Salt	Peak Height SIMV	SIMV-Sodium Salt Peak Height/SIMV Peak Height
SIMV	1	6,661,164.185	12,978,716.510	0.513
RIG/SIMV	1	389,934.251	68,640.012	5.680

**Table 4:** Brillouin peak position and linewidth of RIG, ALG and SIMV as obtained by DHO fits (see Materials and Methods). These data represent the average of several measurements made on both bare RIG/SIMV and covered RIG/SIMV/ALG scaffolds.

Component	Peak Position (GHz)	Linewidth (GHz)
RIG	35.6 ± 0.7 39.0 ± 0.3	4.2 ± 0.6 2.7 ± 0.7
ALG	23.1 ± 0.5	1.9 ± 0.1
SIMV	14.5 ± 0.4	3.0 ± 0.6

## Data Availability

The original contributions presented in the study are included in the article/Supplementary Material. Further inquiries can be directed to the corresponding authors.

## Supplementary Materials

The following supporting information can be downloaded at: https://www.mdpi.com/article/10.3390/jfb17090461/s1, Figure S1: Image of RIG/SIMV/ALG_2_ scaffolds obtained by optical microscopy (with a 10 × 0.25 resolution).
