# Supplementary material for: Effect of Alginate Coatings on Hydroxyapatite/β-Tricalcium Phosphate Scaffold Behavior
Source: J Funct Biomater. 2026 Sep 8;17(9):461. doi: 10.3390/jfb17090461 (PMC13608615; doi:10.3390/jfb17090461)
Supplement: Supplementary file 1 [file jfb-17-00461-s001.zip › jfb-4486989-supplementary.pdf]

SUPPLEMENTAL INFORMATION

Effect of Alginate coatings on hydroxyapatite/β-tricalcium phosphate scaffolds behaviour

Michela Piccinini[1], Maria Laura Belladonna[2*], Chiara Suvieri[2], Sara Meoni[1], Daniela Lanari[1], Silvia Caponi[3], Francesco Bonacci[4], Maurizio Ricci[1], Alessandro Di Michele[4], Valeria Ambrogi[1]

[1] Department of Pharmaceutical Science, University of Perugia; Italy, michela.piccinini@dottorandi.unipg.it, sara.meoni00@gmail.com; daniela.lanari@unipg.it; maurizio.ricci@unipg.it; valeria.ambrogi@unipg.it.

[2] Department of Medicine and Surgery, University of Perugia; Italy, marialaura.belladonna@unipg.it; chiara.suvieri@unipg.it.

[3] Istituto Officina dei Materiali, National Research Council (IOM-CNR), Unit of Peru-gia, c/o Department of Physics and Geology, University of Perugia, Italy; silvia.caponi@cnr.it.

[4] Department of Physics and Geology, University of Perugia, Italy; francesco.bonacci@unipg.it; alessandro.dimichele@unipg.it.

[*] Correspondence: valeria.ambrogi@unipg.it; 0039 075 5855125, 06123 Perugia, Italy (V.A.); marialaura.belladonna@unipg.it, 0039 075 5858244 (M.L.B.)

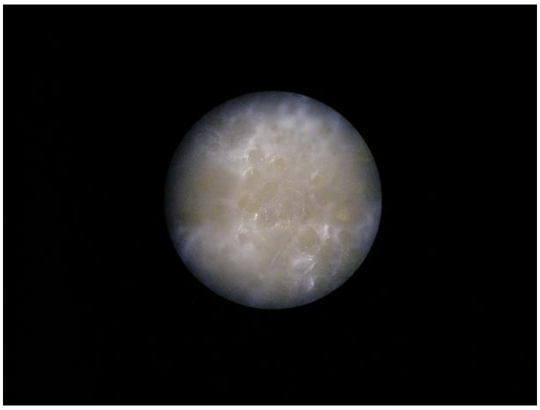

Figure S1. Images of RIG/SIMV/ALG_2 scaffolds obtained by optical microscopy (with a 10 x 0.25 resolution).
